# Shared and Diverging Neural Dynamics Underlying False and Veridical Perception

**DOI:** 10.1523/JNEUROSCI.1479-24.2025

**Published:** 2025-06-05

**Authors:** Joost Haarsma, Dorottya Hetenyi, Peter Kok

**Affiliations:** Department of Imaging Neuroscience, UCL Queen Square Institute of Neurology, University College London, London WC1N 3AR, United Kingdom

**Keywords:** decoding, hallucinations, MEG, oscillations, predictive coding

## Abstract

We often mistake visual noise for meaningful images, which sometimes appear as convincing as veridical percepts. This suggests considerable overlap between the mechanisms that underlie false and veridical perception. Yet, false percepts must arise at least in part from internally generated signals. Here, we apply multivariate analyses to human MEG data to study the overlap between veridical and false perception across two aspects of perceptual inference: discrimination of content (what did I see?) and detection (did I see something?). Male and female participants performed a visual discrimination task requiring them to indicate the orientation of a noisy grating, as well as their confidence in having seen a grating. Importantly, on 50% of trials, only a noise patch was presented. To exclude external signals driving false percepts, noise patches were carefully designed not to contain orientation signal. Still, participants occasionally confidently reported seeing a grating on noise only trials, i.e., false percepts. Decoding analyses revealed a sensory signal reflecting the content of these false percepts, despite no such grating being physically presented. Uniquely, high confidence false, but not veridical, percepts were associated with increased prestimulus high alpha/low beta [11−14 Hz] power, potentially reflecting enhanced reliance on top-down signaling on false percept trials. Later on, a shared neural code reflecting confidence in stimulus presence emerged for both false and veridical percepts. These findings suggest that false percepts arise through neural signals reflecting both sensory content and detection, similar to veridical percepts, with an increase in prestimulus alpha/beta power uniquely contributing to false percepts.

## Significance Statement

The neural mechanisms underlying false percepts are likely different from those that underlie veridical perception, as the former are generated endogenously, whereas the latter are the result of an external stimulus. Yet, false percepts often get confused for veridical perception, suggesting a converging mechanism. This study explores the extent to which the mechanisms diverge and converge. We found that both high confidence false and veridical percepts were accompanied by content-specific stimulus-like orientation signals, as well as a shared signal reflecting perceptual confidence. In contrast, we found that false, but not veridical, percepts were preceded by increased high alpha/low beta [11–14 Hz] power, possibly reflecting a reliance on endogenous signals.

## Introduction

When we try to make sense of our noisy visual surroundings, we sometimes mistake our own internally generated signals for externally caused sensations. For example, a cat owner may have such a strong expectation to see their pet that they misperceive a blurry flash in their visual periphery for it. Despite these false perceptual experiences relying on internally generated signals, they sometimes appear to be just as real as veridical percepts, suggesting at least partially overlapping neural mechanisms between veridical and false perception. However, the extent to which the underlying neural mechanisms are similar remains unclear.

Perceptual inference has two main aspects, namely, inference about the content of an experience (what did I perceive?), as well as an inference about the presence of this experience (did I perceive it?). Indeed, some recent empirical and theoretical work has highlighted the importance of separating these distinct and discrete stages of inference (Fleming, 2020; Mazor et al., 2020; Mazor and Fleming, 2020). Therefore, false inferences might arise from content or detection related signals. In line with the latter, one previous study revealed neural signals reflecting confidence in false percepts, but not their specific contents (Mostert et al., 2015). Further, recent studies have revealed that false percepts can be accompanied by sensory-like signals reflecting falsely perceived low-level stimuli (Haarsma et al., 2023), as well as incorrectly recognized objects (Alilović et al., 2023), suggesting that false percepts can arise from the same signals that underpin veridical sensory processing. In the present study we will investigate both the detection and sensory signals underlying false inference in parallel during a perceptual discrimination task.

While false and veridical percepts may share considerable overlap, their neural origins must be separate in some sense, as they rely on different neural inputs by definition—the former being generated internally and the latter externally. Prestimulus oscillatory dynamics are a candidate mechanism for a point of divergence. Previous studies have consistently linked a decrease in alpha power to increased hit rates by inducing a more liberal detection threshold, while not affecting sensitivity, possibly through increasing neuronal excitability (Ergenoglu et al., 2004; Van Dijk et al., 2008; Achim et al., 2013; Chaumon and Busch, 2014; Mathewson et al., 2014; Boncompte et al., 2016; Benwell et al., 2017; Iemi et al., 2017; Samaha et al., 2017, 2020; Brüers and VanRullen, 2018; Iemi and Busch, 2018). Yet, evidence linking decreased alpha to increased false percepts remains mixed (Lange et al., 2013; Keil et al., 2014). In contrast, a second body of work has linked increased alpha and beta to top-down cognitive processes believed to contribute to false percepts. For example, increased beta power plays a role in interpreting ambiguous stimuli in the language (Iversen et al., 2009) and visual domains (Okazaki et al., 2008; Hipp et al., 2011), as well as mental imagery (Bartsch et al., 2015; Villena-González et al., 2018) and perceptual predictions (Engel et al., 2001; Bastos et al., 2012, 2020; Fujioka et al., 2012; Sherman et al., 2016; Auksztulewicz et al., 2017; Turner et al., 2023). Thus, if top-down mechanisms play a role in driving false percepts, we might find enhanced power in the alpha/beta frequency bands to be related to false percepts.

Here, we used multivariate decoding techniques in combination with MEG to study the overlap between false and veridical perception at the content and detection-level, as well as to study a potentially unique role for neural oscillations in driving false percepts. To preview, false percepts resembled veridical perception to the extent that both were associated with stimulus-like content-specific signals reflecting the perceived gratings, as well as a shared signal reflecting confidence in stimulus presence. Uniquely however, false, but not veridical, percepts were associated with an increase in prestimulus high alpha/low beta (11–14 Hz) power, possibly reflecting endogenous feedback signals. Together, these findings show that false percepts arise from sensory-like neural mechanisms, similar to veridical perception.

## Materials and Methods

### Ethics statement

This study was approved by the University College London Research Ethics Committee (R13061/RE002) and conducted according to the principles of the Declaration of Helsinki. All participants gave written informed consent prior to participation and received monetary compensation (£7.50 an hour for behavioral training, £10 an hour for MEG).

### Participants

Twenty-five healthy human volunteers with normal or corrected-to-normal vision participated in the MEG experiment. Two participants were excluded due to missing trigger data. The full sample was used to analyze behavioral effects, while the imaging sample contained the remaining 23 participants (22 female; age 25 ± 4 years; mean ± SD).

### Stimuli

Grayscale luminance-defined sinusoidal grating stimuli were generated using MATLAB (MathWorks, RRID:SCR_001622) and the Psychophysics Toolbox (Brainard, 1997). During the behavioral session, the stimuli were presented on a PC (1,024 × 768 screen resolution, 60 Hz refresh rate). During the MEG recording session, stimuli were projected onto a screen in front of the participant (1,920 × 1,200 screen resolution, 60 Hz refresh rate). On grating-present trials (50%), auditory cues were followed by a grating after a 750 ms delay (0.5 cpd spatial frequency, 33 ms duration), displayed in an annulus (outer diameter, 10° of visual angle; inner diameter, 1°; contrast decreasing linearly to 0 over 0.7° at the inner and outer edges), surrounding a fixation bull’s-eye (0.7° diameter). These stimuli were combined with one of four noise patches, which resulted in a 4% contrast grating embedded in 20% contrast noise during the MEG session. This was done by simply multiplying the grating matrix with 0.04 and adding it to a noise matrix multiplied by 0.2. On noise-only trials, one of the four noise patches was presented on its own. Noise patches were created by smoothing pixel-by-pixel Gaussian noise with a Gaussian smoothing filter, ensuring that the spatial frequency of the noise matched the gratings (Wyart et al., 2012). This was done to ensure that the noise patches and gratings had similar low-level properties, increasing the likelihood of false percepts (Pajani et al., 2015). Previous studies have found that fluctuations in orientation energy in noise patches can contribute to false percepts (Gosselin and Schyns, 2003; Smith et al., 2012; Wyart et al., 2012; Weilnhammer et al., 2024). In the present study, we explore the question whether false percepts can be induced by internally generated signals alone. Therefore, to avoid including noise patches which contained grating-like orientation signals by chance, 1,000 noise patches were processed through a bank of Gabor filters with varying preferred orientations. Only noise patches with low (2%) signal energy for all orientations were selected to be included in the present experiment. The resulting four noise patches were used for all trials throughout the experiment, in a counterbalanced manner, ensuring that the specific orientations reported on these false percepts trials could only have been triggered by internal mechanisms (Pajani et al., 2015; Haarsma et al., 2023). Follow-up analyses showed that neither noise patch was consistently associated with a perceptual bias and neither of the four noise patches induced orientation-specific activity in the MEG signal, confirming the success of this approach. During the practice session on the first day, the contrast of the gratings was initially high (80%), gradually decreasing to 4% toward the end of the practice. The central fixation bull’s-eye was present throughout the trial, as well as during the intertrial interval (ITI; randomly varied between 1,000 and 1,200 ms). The auditory cues consisted of two 200 ms tones of 450 and 1,000 Hz, respectively, referred two as the low and high tone.

### Experimental procedure

Participants were required to perform a visual perceptual discrimination task. Trials consisted of an auditory expectation cue, followed by a grating stimulus embedded in noise on 50% of trials [750 ms stimulus onset asynchrony (SOA) between cue and grating]. The auditory cue (high or a low tone) predicted the orientation of the grating stimulus (45° or 135°) on grating-present trials. On these grating-present trials, a grating with the orientation predicted by the auditory cue was presented embedded in noise, while on noise-only trials (50%) only a noise patch was presented. The stimulus was presented for 33 ms. After the stimulus disappeared, the orientation response prompt appeared, consisting of a left and right pointing arrow on either side of the fixation dot (location was counterbalanced). Participants were required to select the arrow corresponding to their answer (left arrow for anti-clockwise, or 135°, right arrow for clockwise, or 45°; 1 s response window) through a button press with their right hand, using either a button box in the MEG or a keyboard during behavioral training. Subsequently the letters “CONF?” appeared on the screen probing participants to indicate their confidence that they had seen a grating (1 = I did not see a grating, 2 = I may have seen a grating, 3 = I probably saw a grating, 4 = I am sure I saw a grating), using one of four buttons with their left hand (1.25 s response window). It is worth highlighting here that confidence here reflected participants’ belief that a grating was present, not their confidence in their orientation report. It is therefore likely highly correlated to metrics like perceptual awareness scales or stimulus visibility, rather than a metric of meta-cognitive confidence in the orientation decision (Sandberg and Overgaard, 2015).

**Figure 1.**
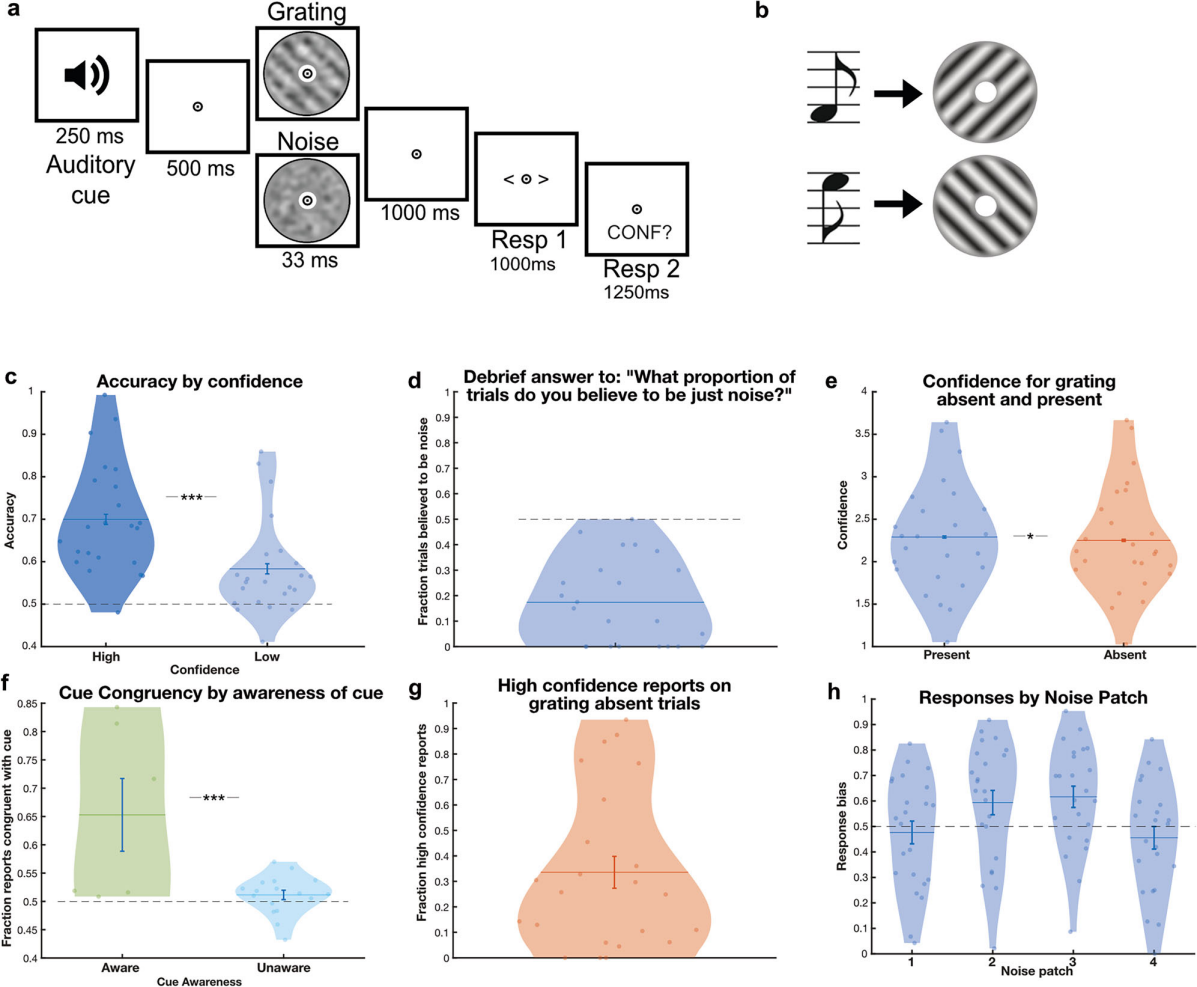
Experimental design and behavioral findings. ***a***, An auditory cue was followed by either a low contrast grating embedded in noise (50% of trials) or a noise patch (50%). Participants indicated which orientation they saw and how confident they were that a grating was presented. ***b***, One sound predicted the appearance of a 45°, or clockwise, oriented grating, while the other predicted a 135°, or anti-clockwise, orientated grating. Auditory cues were 100% valid on grating-present trials. ***c***, Participants were more accurate on high confidence trials. ***d***, Upon debriefing participants believed that only 18% of trials contained noise, compared with the real number of 50%. ***e***, Participants were more confidenct in having seen a grating on present trials compared with absent trials. ***f***, Participants were driven by the cue on noise-only trials when they were aware of the cues meaning. ***g***, On noise-only trials, participants reported high confidence in having seen a grating on 35% of trials. ***h***, None of the four noise patches that were used were associated with a significant perceptual bias (all *p* > 0.05).

On the first day of testing, participants took part in a behavioral practice session. The practice session consisted of an instruction phase with seven blocks of 16 trials where the task was made progressively more difficult by decreasing the contrast of the grating while leaving the noise contrast unchanged, effectively reducing its visibility. During the training, verbal and written instructions were provided. During these practice runs, the auditory cues predicted the orientation of the grating stimulus with 100% validity (45 or 135°; no noise-only trials). After the completion of the instructions, the participants completed four runs of 128 trials each, separated into two blocks of 64 trials each. In the first two runs, the expectation cues were 100% valid, to ensure participants learnt the association, while in the final two runs the cues were 75% valid (i.e., the grating had an unexpected orientation on 25% of trials), to test whether participants might have adopted a response bias. Grating contrast decreased over the four runs, specifically the contrast levels were 7.5, 6, 5, and 4%, while the contrast of the noise patches remained constant at 20%. No noise-only trials were presented on Day 1. On the second day, participants performed the same task during the MEG recording. During this session, eight runs were completed, each consisting of 64 trials. This time the grating contrast was fixed at 4% on grating-present trials, and on 50% of the trials the gratings were omitted and only noise patches were presented, resulting in noise-only trials (Fig. 1*a*). On grating-present trials, the cues always predicted the orientation of the grating with 100% validity (Fig. 1*b*). On noise-only trials, the cue was irrelevant, since no grating was presented. After each run, a localizer run followed where gratings oriented either 45 or 135° were presented while participants performed a distracting fixation dimming task. The purpose of this localizer run was to uncover an orientation-specific MEG signal by conducting a linear discriminant analysis (LDA) on the localizer trials, which were then generalized to the main experiment to uncover sensory signals underlying false percepts. Each run lasted ∼8 min, totaling ∼64 min.

### Preprocessing of MEG data

MEG was recorded continuously at 600 samples/second, using a whole-head 273 channel axial gradiometer system (CTF Omega, VSM MedTech), while participants sat upright. A photodiode was used to measure the onset of the visual stimuli through the presentation of a small white square in the bottom-right corner of the screen on both localizer trials and main experiment trials. This was done to ensure that the trials were aligned exactly to stimulus presentation. Note that the white square was not visible to participants, as it was covered by the electrode. The first experimental run (out of eight) for each subject was removed as data quality tended to be poor while the participant was acclimatizing to the experimental setting. This left seven runs for analyses. Trials were segmented 3,000 ms prestimulus and 3,000 ms poststimulus. Movement and eyeblink artifacts were manually selected and inspected before being rejected from the data. Independent component analyses were applied to the complete dataset to identify components that reflect eyeblinks as well as cardiac related signals, which were manually inspected and removed from the data for each subject. The MEG data for the main experiment were baseline corrected from 1,000 to 750 ms prestimulus, so as not to include the time window between the onset of the cue and the onset of the stimulus, which was a time window of interest. The MEG data for the localizer were baseline corrected from 200 to 0 ms prestimulus.

### ERF analyses

ERF analyses were conducted for exploratory reasons. We tested for differences in ERF amplitude for high and low confidence trials. Cluster-based analyses where then conducted on the sensor level using Monte-Carlo permutation tests (*N* = 10,000), at a significance threshold of *p* < 0.05 for the initial threshold for determining a significant difference in ERF as well as for determining significant clusters. ERFs were computed separately for high and low confidence trials, for both grating-present and noise-only trials.

### Frequency analyses

Frequency power was estimated across all 273 MEG channels from 2,000 ms prestimulus to 2,000 ms poststimulus in steps of 50 ms, for the frequencies 2–30 Hz with steps of 1 Hz, using Morlet wavelets (width, 7). To test for prestimulus changes in alpha and beta power, we used cluster-based permutation tests with 10,000 iterations, at a significance threshold of *p* < 0.05, while averaging over the alpha band (8–12 Hz) or the beta band (13–20 Hz). The frequency analyses were conducted across the topography of all 273 channels and were corrected for multiple comparisons across time and space (channels) through spatiotemporal cluster permutation tests (Maris and Oostenveld, 2007). The alpha and beta frequency bands were tested separately and therefore further corrected for multiple comparisons through Bonferroni’s correction (8–12 Hz and 13–20 Hz, respectively). We furthermore conducted post hoc *t* tests to find the exact frequency bands driving the effect by repeating the analyses for each frequency between 8 and 20 Hz with steps of 1 Hz. For prestimulus effects, we tested the time window from the onset of the cue to the onset of the stimulus, i.e., −750 ms to 0 ms. For poststimulus effects, the time window of interest ranged from 0 to 1,000 ms. We conducted additional exploratory analyses that included the full prestimulus time window −2,000 ms and full poststimulus time window. Note that these time windows include the onset of the auditory stimulus (−750 ms) and the orientation response cues (1,000 ms).

### Decoding analyses

We decoded both stimulus content (grating orientation) and participants' confidence in having seen a stimulus from the MEG signal using a two-class LDA ( Mostert et al., 2015). For content decoding, we were interested in testing whether subjectively perceived orientations were accompanied by sensory-like orientation signals, on both false percept and veridical trials. To obtain a measure of a sensory orientation signal, we trained a decoder on MEG data from separate localizer blocks to distinguish the orientation of clearly presented (but task-irrelevant) gratings. Localizer blocks were presented at the end of each run (of which there were eight), which each contained 80 trials of clearly presented gratings. Right- and left-tilted orientations were presented equally, ensuring that the training data for the localizer was balanced. Once trained, we applied this decoder to the main experiment, to test for orientation-specific neural activity on false percept and veridical trials. To train the decoder, the 273 MEG channels were used as features. To confirm the ability of the decoder to retrieve orientation signals, we also trained and tested it within the localizer data using a leave-one-out procedure, where all localizer blocks except one served as the training data to decode the remaining block. This procedure was repeated for all blocks. Time points were averaged across a moving time window containing 17 samples [(17/600) = 28.3 ms], with steps of three samples [(3/600) = 5 ms]. The covariance matrix was taken into account in the decoder as recommended in previous studies to address correlations between neighboring MEG sensors (Brouwer and Heeger, 2009; Mostert et al., 2015). For details on the implementation of the decoder, see Mostert et al. (2015). The output of the decoder optimally discriminates between the two classes it is trained on. If there is information in this channel, we expect the mean amplitude for class 1 to be higher than that of class 2. In contrast if there is no information in the signal, the difference should be 0. In other words, the difference in the decoder output between classes is a measure of the discriminability of these classes based on neural signals. Note that we deviated here from a more standard discrimination analyses, where each trial or time point is assigned a categorical label based on whether evidence is in favor of class 1 or 2. In this instance, we preserved the continuous output, rather than binarize it, as this preserves more information. We refer to this metric as “decoder output” in our figures.

For the purpose of decoding confidence in having seen a stimulus, we used a cross-validated decoding procedure similar to the approach used to decode orientations within the localizer blocks. Specifically, using a leave-one-block-out procedure, we decoded confidence in having seen a grating during the main experiment, by training the LDA on binarized confidence response, such that the confidence responses reflected higher or lower than average confidence. This average was calculated on an individual level, to ensure sufficient numbers of trials in the high and low confidence conditions. For the remainder of the paper, high and low confidence will thus mean higher or lower than average for each specific subject. The exception is Figure 1*g* where we visualize the variation in high confidence false alarms across participants. Further, to ensure balanced training data, a random set of trials of the overrepresented condition was selected equal to the number of trials in the underrepresented condition. This led to a few participants for whom we deemed there was not enough data to train the discriminator (one on all trials, four on grating-present trials, seven on noise only). All other parameters of the LDA were identical to the orientation decoding analysis.

To test for sensory-like orientation-specific activity during the main experiment, we trained on the group-level peak of the localizer decoding and averaged over a 10 ms time window (100–110 ms). Generalizing this signal to the main experiment resulted in a trial-by-trial estimates of relative evidence in favor of a left titled grating over the course of a trial. Critically, we subtracted trials where participants reported right tilted gratings from left titled grating trials. If there is orientation-specific information in the MEG signal reflecting the reported stimulus, this should result in a positive signal. Subsequently, low confidence trials were subtracted from high confidence trials to show activity specific for orientations that the participant indicated to have seen. Then, cluster-based permutation tests were conducted on the time courses of these signals (Maris and Oostenveld, 2007). In the first step of the permutation test, clusters were defined by adjacent points that crossed a significance threshold of *p* < 0.05. The number of permutations for the temporal generalization matrix was limited to 1,000 due to the large sample space. In contrast, when performing cluster-based analyses on a single time course, as when we generalized from the localizer to the main experiment, or the diagonal of the temporal generalization matrix for the confidence decoding 10,000 permutations were used. A cluster in the true data was considered significant if *p* < 0.05 based on the null distribution generated by the permutations. The time window for the confidence decoding was initially −1,000 to 2,000 ms relative to stimulus onset to include the time window where the orientation responses were given (1,000–2,000 ms). For the analyses pertaining to finding content-specific signals, we focused on pre- and poststimulus time windows separately and excluded the time window where the orientation was reported (i.e., 1,000 ms onward). We performed a number of follow-up analyses for the confidence decoding. These follow-up analyses involved cross-decoding between noise-only and grating-present trials, to explore whether they shared a neural code for perceptual confidence. Furthermore, we performed control analyses where we balanced the number of trials in the high and low perceptual confidence conditions by subsampling to the condition with the fewest trials, to ensure that the effects were not driven by an overrepresentation of a single condition.

### Source localization

To visualize the source of the confidence decoding, we performed source localization analyses. We did not collect individual anatomical MRI scans for our subjects; instead, a template MRI and default head and source models as present in the FieldTrip toolbox (www.fieldtriptoolbox.org) were used (see below; Oostenveld et al., 2011). Previous studies have demonstrated that little anatomical specificity is lost using a group-based template approach (Holliday et al., 2003).

The spatial pattern that underlies classification in a linear discrimination analysis is driven by the difference in magnetic fields between the two conditions on which the decoding is based. Therefore, one can visualize the source of a decoder by estimating the sources of the two different conditions and compute the difference (Haufe et al., 2014). For our purposes, we computed the absolute difference within the 1,000–2,000 ms time window (where decoding was strongest), divided by the source map of the low confidence condition, thereby estimating percentage signal change. Taking the absolute difference will visualize which source signals are involved in perceptual confidence, without making assumptions about the sign of the signal.

We also performed source localization on the frequency analyses. Here, there was a direct translation from the univariate effects to the underlying source map, as they seek to refute the same null hypothesis. We therefore computed cluster-based statistics on the −750–0 ms time window for the difference in 13 Hz (±1.5 Hz) power between high and low confidence false percept conditions in source space. The same parameters were used as on the sensor level (10,000 permutations, cluster-defining threshold: *p* = 0.05, cluster-level threshold: *p* = 0.05).

For both the frequency and decoding source localization, we used the default forward and source models from the FieldTrip toolbox which were then warped to participants’ specific fiducials based on the MEG sensors. The spatial filter was computed for the time windows of interest in the averaged data, which was subsequently applied separately to the two conditions of interest (high and low confidence trials). Source localization was then computed for the two conditions of interests. For the confidence decoding, a percentage absolute signal change was computed in source space, whereas for the frequency analyses *t* maps were visualized for significant clusters.

### Behavioral analyses

We tested participants’ accuracy scores, as well as the relationship between accuracy and confidence in stimulus presence. We further tested whether participants were significantly biased by the auditory cues on noise-only trials and performed post hoc tests to see whether these effects were driven by cue awareness. All tests were conducted within subject and two sided. Statistics were conducted in JASP (JASP Team, 2023).

## Results

### Participants experienced false percepts that were independent of perceptual expectation cues

Participants performed a grating discrimination task under noisy conditions, while on 50% of the trials noise-only patches were presented. Participants indicated both the perceived orientation and their degree of confidence in having perceived a grating (regardless of orientation). Participants accurately identified the grating orientation on grating-present trials more often than expected by chance (mean accuracy = 0.65, SD = 0.12, T{22} = 6.5, *p* < 0.001). Furthermore, they were more accurate when they were confident that they had seen a grating (i.e., higher than average confidence across trials) than when they were not (high: mean = 0.70, SD = 0.13; low: mean = 0.58, SD = 0.11; paired *t* test; T{22} = 6.89, *p* < 0.001; Fig. 1*c*), demonstrating that they were able to perform the task and used the confidence ratings in a meaningful way. It is worth repeating that here participants reported their confidence in having seen a grating, rather than confidence in their orientation report, and thus effectively gave a perceptual awareness response (Sandberg and Overgaard, 2015). Upon debriefing, all participants but one underestimated the frequency of noise-only trials, believing on average that 0.17 (SD = 0.17) of trials contained just noise, while the true proportion was 0.50 (Fig. 1*d*). Participants were slightly more confident on grating-present trials (mean confidence = 2.26, SD = 0.69, on a scale of 1–4) than noise-only trials (mean = 2.21, SD = 0.66) (T{22} = 2.58, *p* = 0.017; Fig. 1*e*). Strikingly, participants reported perceiving a grating with high confidence on 34% of noise-only trials (Fig. 1*g*). The perceptual expectation cues significantly biased which orientation participants perceived on noise-only trials (0.55 false percepts congruent with the cue, chance level is 0.50, T{22} = 2.26, *p* = 0.034). This effect was driven by the individuals who became aware of the meaning of the cues (*N* = 6 out of 23; Fig. 1*f*), potentially reflecting a response bias. Indeed, those aware of the cue had significantly stronger effects of the cue (T{21} = 3.61, *p* < 0.0016). Indeed, the unaware group on their own was not significantly affected by the cue (T{16} = 1.38, *p* = 0.19). High confidence false percepts were not more affected by the cues than low confidence percepts, i.e., guesses (T{22} = 0.99, *p* = 0.33). The noise patches were designed such that they did not contain any orientation-specific signals. However, we nevertheless tested whether participants showed a systematic bias toward either of the orientations for any of the four noise patches. This revealed that only one out of the four showed a trend-level bias (*p* = 0.056, other *p* > 0.1; Fig. 1*h*). In sum, these results indicate that participants regularly had false grating percepts but that these were hardly, if at all, affected by the predictive cues or the specific noise patches. This is highly consistent with a previous study using the same experimental paradigm (Haarsma et al., 2023).

### Orientation-specific activity reflecting the perceived stimulus was present both pre- and poststimulus on high confidence false percept trials

**Figure 2.**
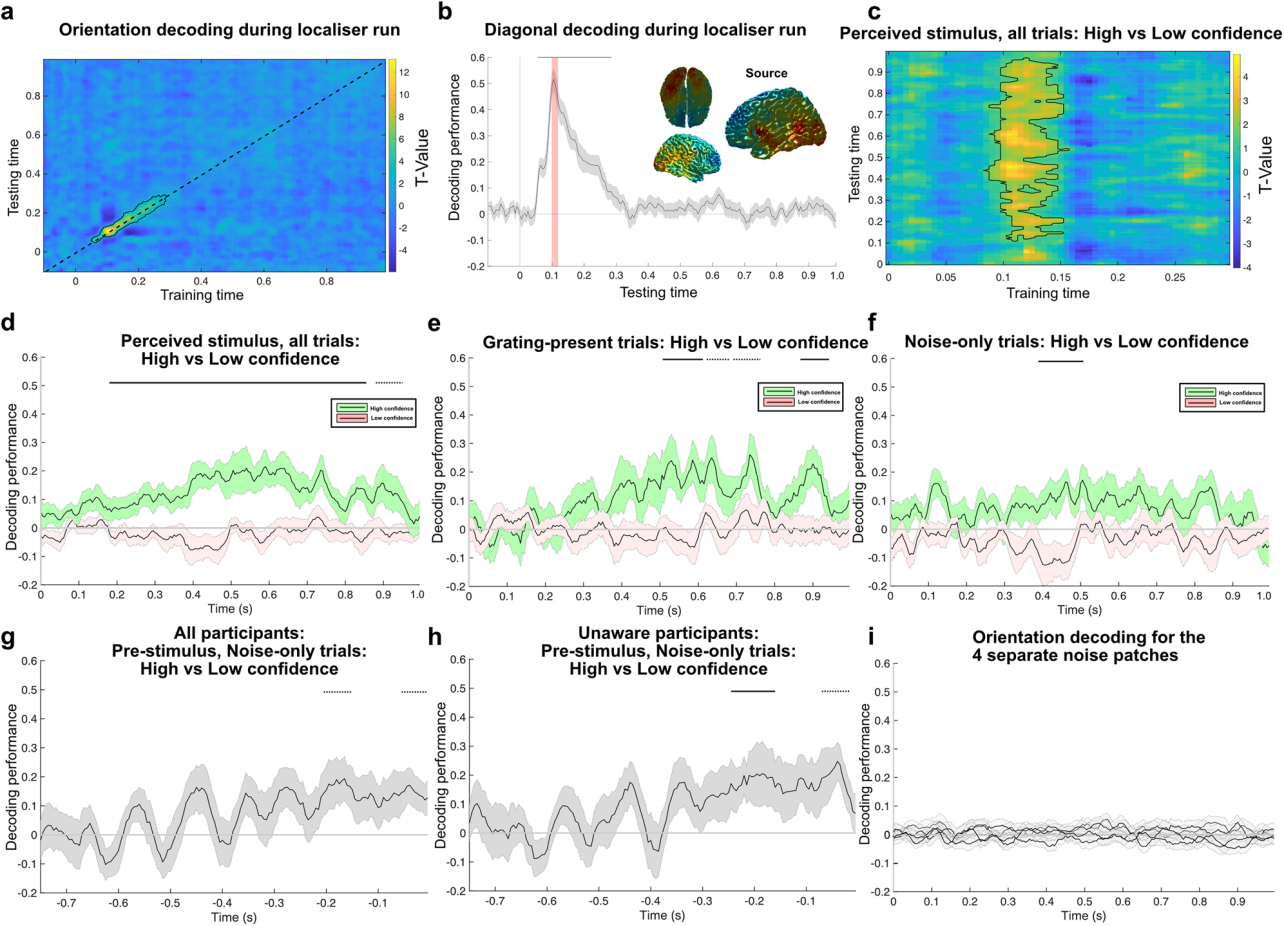
Decoding of perceived orientation. A linear discriminator analyses was used to decode orientation-specific activity from a functional localizer, which was then generalized to the main experiment. The orientation of a clearly presented grating could be decoded from the functional localizer with strong clusters in the temporal generalization matrix (***a***) and the diagonal (***b***). ***b***, The peak of the localizer window that served as the training time point for the generalization to the main experiment is indicated in shaded red. Orientation decoding was primarily driven the occipital lobe. ***c***, Generalizing from the localizer to the main experiment revealed a clear signal reflecting the perceived orientation across all trials, specific to the 100–150 ms training time window on the localizer. ***d***, Training on the peak of the localizer decoding (100–110 ms) and separating on high and low confidence trials reveals a clear orientation-specific signal on high confidence trials that is absent on low confidence trials. ***e***, The same pattern was found when separating trials on grating-present (***e***) and noise-only trials (***f***). Thick lines represent significant clusters (*p* < 0.05), dotted lines reveal almost-significant clusters (*p* < 0.1). Training on the localizer and generalizing to the prestimulus time window revealed orientation-specific activity reflecting subsequent false percepts (***g***), which is significant in unaware participants (***h***). ***i***, We tested the hypothesis that the noise patches themselves induced orientation-specific activity, which we found no evidence for.

In order to test the hypothesis that false percepts arise from sensory-like processes, we performed an LDA to decode the orientation of high contrast, task-irrelevant gratings presented in a separate localizer block in order to then test for the presence of these signals during the main experiment. First, we confirmed that the orientation of these high contrast gratings could be decoded from the MEG signal (Fig. 2*a*). Cluster-based statistics on the diagonal of the temporal generalization matrix revealed significant decoding from 55 to 270 ms poststimulus (*p* < 0.001, Cohen's *d* = 1.87), peaking at 105 ms (Fig. 2*b*). Further, source localization revealed that the occipital lobe contributed primarily to the decoding of the grating orientation (Fig. 2*b*). We then generalized from the localizer to the main experiment to see whether sensory signals contributed to high confidence false percepts (Fig. 2*c*). Here, we trained the decoder on a 10 ms window centered on the peak of the localizer decoding (100–110 ms). We subtracted orientation-specific stimulus traces on low confidence trials from high confidence ones, for both grating-present and noise-only trials. We focused on the pre- and poststimulus time windows separately (−750 to 0 ms and 0–1,000 ms, respectively). First, across all trials we found a clear orientation-specific neural signal for high versus low confidence trials during the poststimulus time window, from 180 to 855 ms (*p* < 0.001, Cohen's *d* = 0.87; Fig. 2*c*,*d*). Note that from the full temporal generalization matrix (Fig. 2*c*), it becomes clear that it is indeed primarily the peak of the localizer decoding that generalizes to the main experiment. This is likely due to the higher SNR at the peak of the localizer decoding signal, optimally discriminating between left and right tilted orientations. Analyzing grating-present and noise-only trials separately revealed orientation-specific signals for grating-present trials from 505 to 610 ms and 865 to 940 ms (*p* = 0.018 and *p* = 0.043, Cohen's *d* = 0.75 and 0.71; Fig. 2*e*) and critically also on noise-only trials from 390 to 510 ms (*p* = 0.014, Cohen's *d* = 0.68; Fig. 2*f*). Further, during the prestimulus time window, there was a close to significant orientation-specific activity on noise-only trials from −55 to −5 ms and −205 to −150 ms (*p* = 0.064 and *p* = 0.067, Cohen's *d* = 0.64 and 0.52; Fig. 2*g*). After removing participants who were aware of the purpose of the cue, a significant effect of prestimulus orientation-specific activity was still present −245 to −160 ms (*p* = 0.032, Cohen's *d* = 0.58) and −70 to −15 ms (*p* = 0.051, Cohen's *d* = 0.72; Fig. 2*h*). Note, because only noise patches with low (2%) signal energy for all orientations were selected to be included in the present experiment, this orientation-specific activity could not have arisen from the noise patch itself. Further, there was no systematic behavior bias to suggest that the noise patches were consistently perceived to contain the same orientation (see above). However, we still conducted a control analysis to see whether the noise patches themselves elicited orientation-specific signals in the 0–1,000 ms time period. There was no significant decoding above chance for either of the four noise patches (Fig. 2*i*). No prestimulus orientation-specific signals were found on grating-present trials. Taken together, false percepts were associated with orientation-specific sensory-like signals in both the prestimulus and poststimulus time windows.

### A shared neural code reflecting confidence in stimulus presence emerged from 250 ms poststimulus

**Figure 3.**
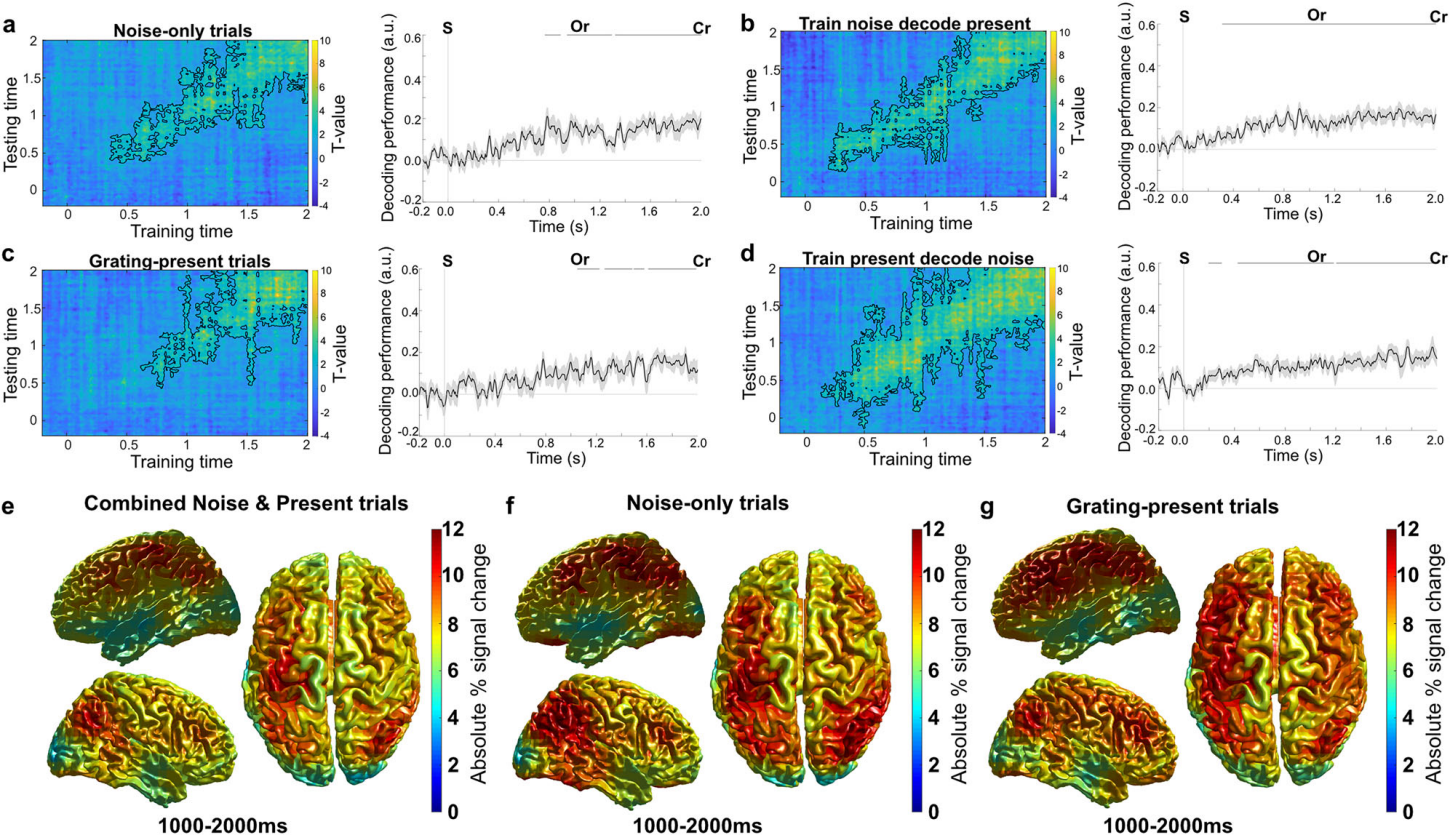
Decoding of perceptual confidence. A linear discriminator analyses was used to identify a perceptual confidence signal. ***a***, A significant confidence signal was found on all trials after stimulus onset. Analyzing noise-only (***b***) and grating-present (***c***) trials separately revealed similar results. Cross-decoding from noise-only to grating-present trials (***d***), and vice versa (***e***), demonstrated that there was a shared neural signal on grating-present and noise-only trials. ***f***, When balancing low and high confidence trial counts, decoding was still successful, demonstrating that the effect was not confounded by imbalanced trial counts. ***g***, Percentage absolute signal change of the high confidence condition compared with the low confidence condition in source localization. Here the combined, noise-only, and grating-present trials in the 1,000–2,000 ms time window are presented separately. Across all three conditions, parietal and frontal cortices contributed most strongly to the linear discrimination analyses. S, onset of stimulus; Or, onset orientation response cue; Cr, onset of confidence response cue.

We next decoded confidence in stimulus presence on both grating-present and noise-only trials using an LDA (Mostert et al., 2015). Rather than training on a separate localizer (during which participants did not give confidence responses), here we used cross-validation to train and test within the main experiment blocks to isolate a signal reflecting participants’ confidence in having seen a grating (regardless of its content). Across both grating-present and noise-only trials, a confidence signal emerged 265 ms poststimulus and was sustained throughout the poststimulus window (265–2,000 ms, *p* < 0.001, Cohen's *d* = 1.6; Fig. 3*a*). This signal was present on both noise-only (765–890 ms, 940–1,295 ms, 1,320–2,000 ms, *p* = 0.03, *p* < 0.01, *p* < 0.001, Cohen's *d* = 0.8, 1.2, 1.3; Fig. 3*a*) and grating-present trials (1,005–1,225 ms, 1,265–1,485 ms, 1,495–1,580 ms, 1,610–1,990 ms, *p* = 0.015, *p* = 0.022, *p* = 0.0095, *p* = 0.001, Cohen's *d* = 0.92, 0.80, 1.46, 1.15; Fig. 3*b*). To test whether the neural representation of perceptual confidence in veridical and false percepts was the same, we trained on perceptual confidence on noise-only trials and decoded the confidence on grating-present trials, and vice versa. Cluster-based analyses revealed a significant effect in the poststimulus time window, demonstrating that the confidence signal on noise-only trials generalized to grating-present trials (310–2,000 ms, *p* < 0.001, Cohen's *d* = 1.55; Fig. 3*c*) and vice versa (195–300 ms, 425–1,185 ms, 1,205–2,000 ms, *p* = 0.034, *p* < 0.001, *p* < 0.001, Cohen's *d* = 0.75, 1.16, 1.20; Fig. 3*d*).

For visualization purposes, we reconstructed the cortical sources of the confidence signal by performing source localization separately on the magnetic fields underlying high and low confidence perceptual reports and computing a signal difference map. This source reconstruction was conducted on the 1,000–2,000 ms time window, where decoding was the strongest. The source map was overlaid on a 3D cortical surface, revealing that the confidence signal emergens from parietal and frontal regions for combined noise and grating present trials (Fig. 3*e*) as well as noise-only (Fig. 3*f*) and grating-present trials (Fig. 3*g*).

### Within experiment cross-validated decoding of perceived orientation

**Figure 4.**
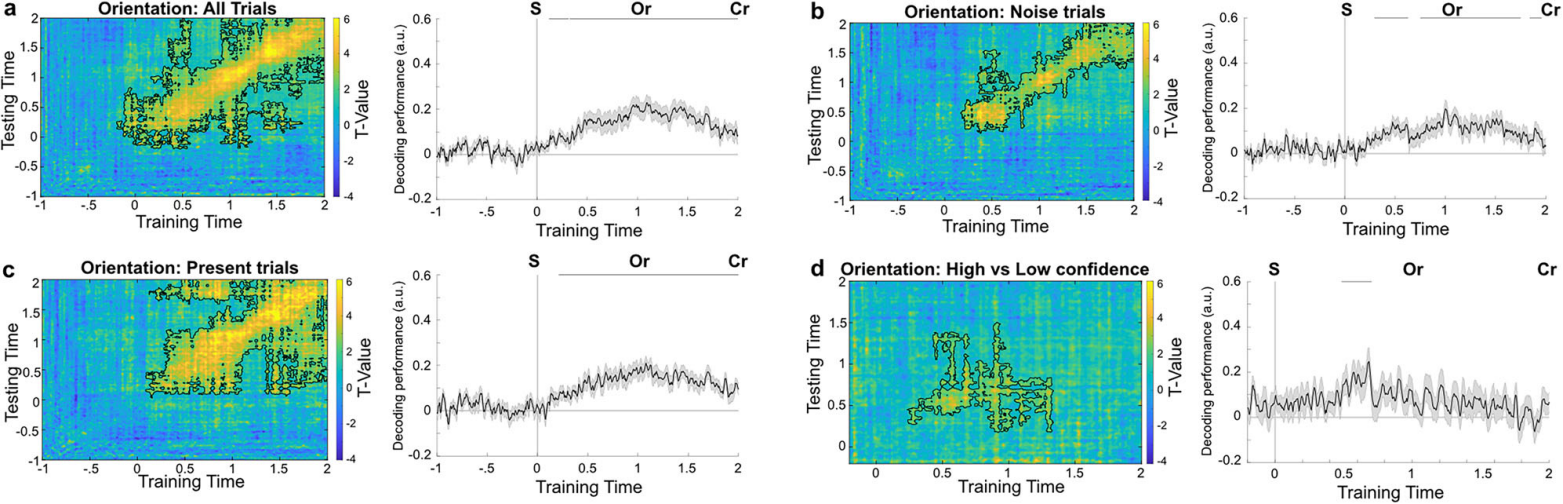
Within decoding of reported orientation. ***a***, As a comparison to the within confidence decoding, we used the same approach to decode the reported orientations during the main experiment. Taking all trials together, we find a signal reflecting the reported percept emerged from 120 to 315 ms poststimulus (*p* = 0.026, Cohen's *d* = 1.02) and from 325 ms until the end of the decoding time window of 2,000 ms (*p* < 0.001, Cohen's *d* = 1.13). ***b***, This signal was present on both noise-only (295–630 ms, 750–1,750 ms, and 1,840–1,960 ms *p* = 0.0018, *p* < 0.001, and *p* = 0.038, Cohen's *d* = 1.03, 0.89, and 0.65, respectively). ***c***, Grating-present trials (210–2,000 ms, *p* < 0.001, Cohen's *d* = 1.43). ***d***, This signal was modulated by participants confidence in stimulus presence, such that decoding was stronger for high confidence trials at 485–705 ms, which overlaps with the time window for sensory-like signals that underlie false percepts (*p* = 0.0019, Cohen’s *d* = 0.77).

Note that the confidence decoding analysis involved cross-validated training and testing within the main experiment, whereas for the orientation decoding analysis we trained on a separate localizer and generalized to the experiment to provide a strong test of the sensory nature of the orientation signals. For completeness' sake, and to aid comparison with confidence decoding, we also applied a within experiment cross-validation approach to decoding the reported orientations (Fig. 4). This analysis will, in contrast to the analyses based on the sensory localizer, also pick up nonsensory-specific signals and thus capture a broader range of processes that might contribute to a participant reporting one orientation over another. Broadly, this confirmed our finding that subjective percepts were reflected by orientation-specific neural signals that were modulated by confidence (Fig. 4*d*). Note, these signals cannot reflect motor responses, as the response mapping was randomized. When decoding participants motor responses, a significant cluster was present exclusively in the response time window (cluster-based analyses on diagonal, all trials: 1,015–2,000 ms, *p* < 0.0001, Cohen's *d* = 2.43), which was not modulated by confidence (*p* > 0.1).

### False percepts were preceded by an increase in prestimulus high alpha/low beta power

**Figure 5.**
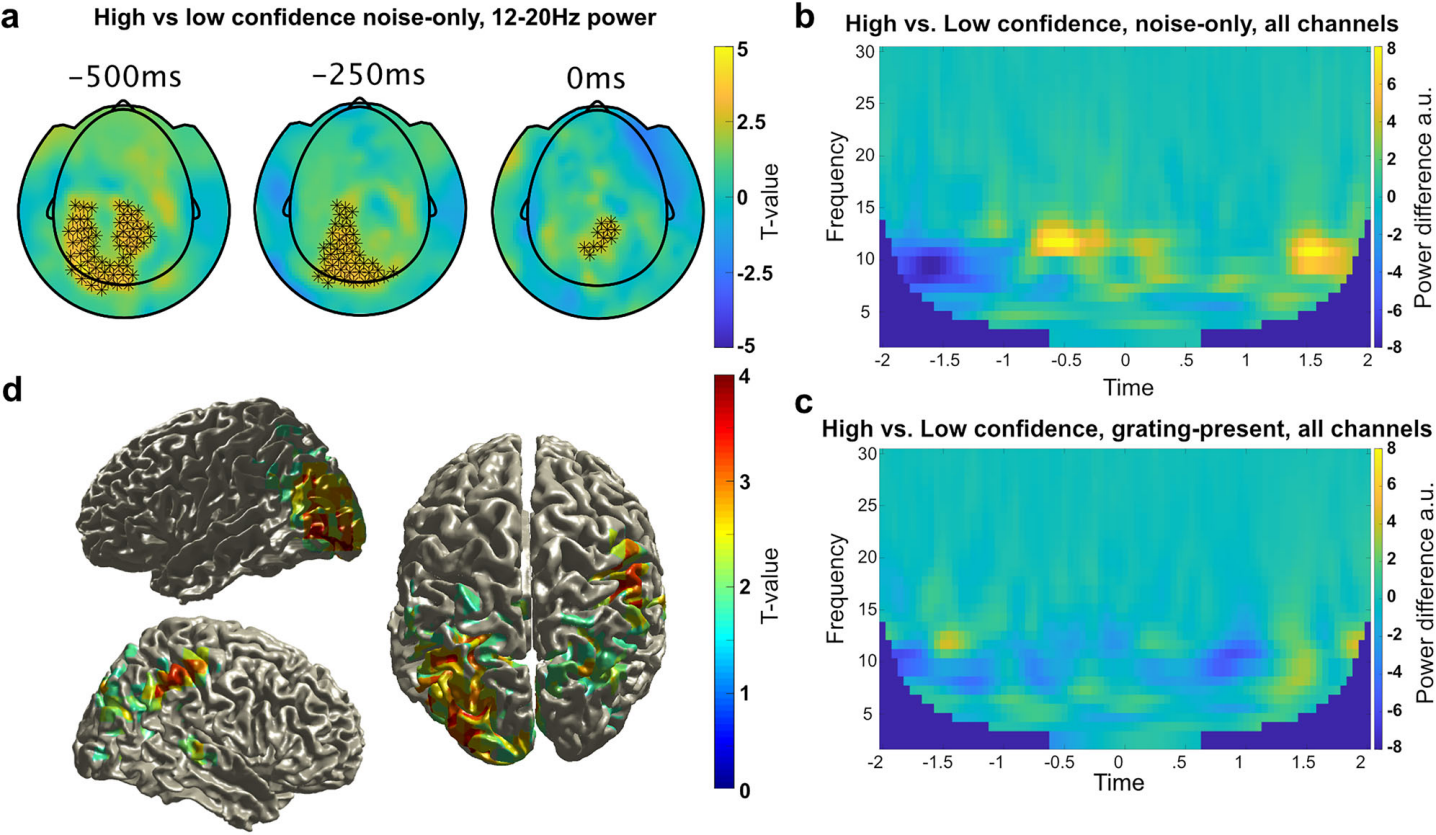
Frequency analyses. ***a***, Scalp topographies of differences in prestimulus beta power for high minus low confidence false percepts. ***b***, Difference in average time × frequency between high and low confidence trials on noise-only trials. ***c***, Difference in average time × frequency between high and low confidence trials on grating-present trials. ***d***, T-map of the difference in source localization for the 13 Hz frequency band ±1.5 Hz between high and low confidence response on noise-only trials. A network of parietal and occipital regions was active prior to high confidence false percepts.

To explore whether prestimulus neural oscillations played a role in driving false percepts, we conducted a time–frequency analyses on the prestimulus time window. Specifically, we tested whether changes in power in the alpha and beta bands preceded high confidence false percepts, that is, trials on which participants indicated high confidence in having seen a grating in the absence of one. We tested for an interaction using a two-way repeated-measures ANOVA with confidence and stimulus presence as two-level factors within the −750 to 0 ms time window (i.e., the time window from the auditory cue to the onset of the stimulus). There was a significant interaction in the beta band (12–20 Hz; −650 to −50 ms, *p* = 0.0039, corrected: *p* = 0.0078), but not in the alpha band (8–12 Hz, −650 to 0 ms, *p* = 0.0939). Post hoc analyses revealed that this interaction was driven by an increase in beta power preceding high confidence false percepts (−650 to 0 ms, *p* = 0.0108, cluster-based permutation test; Fig. 5*a*).

We conducted post hoc analyses to estimate the exact frequencies that drove these effects by repeating the analyses in steps of 1 Hz. This revealed the interaction between stimulus presence and confidence started in the high alpha band (11 Hz, −700 to −300 ms, *p* = 0.0499; 12 Hz, −700 to 0 ms, *p* = 0.002) and continued into the beta band (13 Hz, −700 to 0 ms, *p* = 0.0080; 14 Hz, −650 to −50 ms, *p* = 0.030), as did the increase in beta power preceding high confidence false percepts (11 Hz, −700 to 0 ms, *p* = 0.0039; 12 Hz, −700 to 0 ms, *p* = 0.0059; 13 Hz, −750 to −150 ms, *p* = 0.0079; 14 Hz, −700 to −400 ms, *p* = 0.049).

Thus, the increase in power preceding high confidence false percepts was in the high alpha, low beta band (Fig. 5*b*). In contrast, no effects were found comparing high and low confidence reports on grating-present trials for either alpha or beta power (*p* > 0.5, Fig. 5*c*). No effects were found in the precue (−2,000 to −1,000 ms) or poststimulus (0–1,000 ms) time window for either false or veridical percepts (*p* > 0.5). In sum, an increase in high alpha/low beta band power preceded high confidence false percepts, but not high confidence veridical percepts.

To further explore the temporal profile of this effect, we extended the time window to −2,000 to 0 ms. This control analysis revealed that the interaction between stimulus presence and confidence on beta power started after the onset of the auditory cue (−650 to −50 ms, *p* = 0.018).

We performed source localization analyses on the difference in beta power prior to high and low confidence false percepts in the −750 to 0 ms prestimulus time window centered on 13 Hz (±1.5 Hz). This revealed that the increase in beta power arose from a network including parietal and occipital regions (Fig. 5*d*).

### ERF analyses

Finally, we performed ERF analyses to explore whether confidence in stimulus presence was reflected in the amplitude of the ERFs on grating-present and noise-only trials. For these purposes, we contrasted high and low confidence trials and computed cluster-based analyses on these differences. There were no effects of confidence on ERF amplitude on the sensor level on either noise-only or grating-present trials (*p* > 0.5).

## Discussion

The present study investigated how false percepts arise from internal signals. During a perceptual discrimination task, high confidence false percepts of oriented gratings occurred on a subset of trials where only a noise patch was presented. Prior to high confidence false percepts, there was an increase in parietal occipital high alpha/low beta power (11–14 Hz), followed by sensory-like orientation-specific signals in the occipital cortex reflecting the falsely perceived stimulus. Further, there was a separate signal reflecting confidence in having perceived a stimulus. These signals overlapped considerably with veridical perception. That is, orientation-specific signals underpinned high confident perceptual reports on grating-present trials, and the confidence signal generalized from noise-only trials to grating-present trials and vice versa. However, the prestimulus increase in high alpha/low beta power (11–14 Hz) was only found prior to false percepts, suggesting a unique contribution of alpha/beta oscillations to false perception.

This study builds on previous work linking false percepts to sensory processes. For example, early studies found that misperception of faces (Summerfield et al., 2006) and grating stimuli was linked to early visual cortex activity (Ress and Heeger, 2003). Further studies found sensory-like signals driving detection of hard to perceive letters (Meijs et al., 2019), as well as false percepts with high confidence (Alilović et al., 2023). Further, prestimulus grating orientation signals predict false alarms (Pajani et al., 2015), which we recently have found to be linked to middle layer activity in the early visual cortex specifically (Haarsma et al., 2023), suggesting that the orientation signals in the present study could have arisen from random fluctuations in bottom-up signals. Here, we extend these findings by using MEG to show that orientation-specific signals were present on false percept trials in the absence of any meaningful input and that prestimulus beta power preceded the occurrence of these false percepts.

Previous studies have found that false alarms can arise from fluctuations in signal energy in noise (Gosselin and Schyns, 2003; Smith et al., 2012; Wyart et al., 2012; Weilnhammer et al., 2024). To exclude this possibility, we generated four noise patches that were devoid of orientation energy. Indeed, no systematic biases were found for the four noise patches (*p* = 0.056, bias: 61%, left-tilted responses, others: 59, 46, and 48%, all *p* > 0.2), and the individual noise patches did not induce any orientation-specific neural activity on their own (Fig. 2*i*). Still, it is difficult to fully exclude the possibility that participants could have deployed selective attention to parts of the noise patch which might contain grating-like signals, which could have driven orientation-specific signals in the MEG. However, as orientation-specific activity on grating-absent trials was also present prestimulus in those who were unaware of the cue, it is more likely that these signals were internally generated, rather than reflecting orientation energy in external stimuli.

A number of internal mechanisms could have driven the sensory-like signals on false percept trials. First, predictive coding theories of hallucinations have suggested that false percepts can arise due to overly strong priors (Teufel et al., 2015; Powers et al., 2017; Haarsma et al., 2020, 2025; Kafadar et al., 2020; Schmack et al., 2021). We aimed to elicit these predictions by training participants on specific tone–orientation relationships prior to the experiment. However, just as in our recent layer fMRI study (Haarsma et al., 2023), the cues did not affect perception unless participants were aware of the meaning of the auditory cues. However, fluctuations in endogenous predictions could still have been at play, which may be reflected by increased prestimulus alpha/beta power. Indeed, previous studies have linked beta power to stimulus predictions (Fujioka et al., 2012; Auksztulewicz et al., 2017; Bastos et al., 2020; Turner et al., 2023), and others have found parietal occipital networks to underlie modulation of sensory activity by sensory priors (Rahnev et al., 2011).

The present study found higher alpha/beta power prior to false percepts. This seems at odds with studies finding a link between low alpha power and increased hit rates and enhanced stimulus responses (Chaumon and Busch, 2014; Mathewson et al., 2014; Boncompte et al., 2016; Benwell et al., 2017; Samaha et al., 2017, 2020; Brüers and VanRullen, 2018). However, previous studies of false perception specifically have found higher—not lower—beta power driving false percepts (Keil et al., 2014; Poorganji et al., 2023). Similarly, low prestimulus BOLD activity (which is inversely correlated with alpha/beta power; Scheeringa et al., 2011, 2016) has also been linked to false percepts (Hesselmann et al., 2010; Pajani et al., 2015). Together, this suggests that different mechanisms may be at play for increased hit rates and false alarms. That is, lower alpha power may increase detection by increasing excitability, while higher alpha/beta power might induce false percepts through top-down signals like attention, imagination, illusory perception, and prediction (Buschman and Miller, 2007; Engel and Fries, 2010; Arnal and Giraud, 2012; De Lange et al., 2013; Sherman et al., 2016; Auksztulewicz et al., 2017; Bastos et al., 2020; Raposo et al., 2023). We offer two interpretations for the specific role of alpha/beta power in our study. First, alpha/beta oscillations may reflect beliefs about stimulus presence. That is, while the specifically cued orientations (e.g., a low tone predicts a left-tilted grating) did not influence which orientation participants reported on high confidence false percept trials, there was likely still a strong expectation of a grating (of either orientation) being present on each trial, as participants were not explicitly told that there would be noise-only trials. This was confirmed by participants grossly underestimating the proportion of noise-only trials upon debriefing. Alternatively, beta oscillations might reflect predictions about specific stimulus content, rather than merely stimulus presence. In line with this, a recent study by our lab found alpha/beta oscillations to reflect perceptual expectations (Hetenyi et al., 2024). Mechanisms other than prediction, such as imagery and choice history (Urai et al., 2019; Dijkstra and Fleming, 2023), may also be at play here. Future studies are needed to arbitrate between these hypotheses.

Beyond a sensory signal reflecting the content of the reported false percepts, we also revealed a signal reflecting the confidence in stimulus presence which emerged 250–300 ms poststimulus. Previous studies have linked confidence to the cortical representation of Bayesian probability distributions, as well as activity in higher order regions like the anterior cingulate and prefrontal cortex encoding confidence (Geurts et al., 2022). Our results are consistent with this interpretation, where stimulus decoding in the occipital cortex was associated with higher confidence (i.e., sharper representation), while a confidence code was present in parietal and frontal regions (Geurts et al., 2022). Cross-decoding between grating-present and noise-only trials reveals that this neural signal was shared between false and veridical percepts. One might argue that this signal reflects motor preparation, potentially due to a facilitated motor response on high confidence trials. However, this is unlikely, since participants were required to provide an orientation response before their confidence response. Additionally, the response mapping for the orientation response was randomized, so that a motor response could not be prepared beforehand, precluding the possibility of a motor preparation signal. Instead, the uncovered neural signal is more likely to track confidence in the presence of a stimulus. Previous studies using variations of perceptual discrimination and detection tasks have reported neural correlates of perceptual confidence signals originating from the dorsolateral and (ventral) medial prefrontal cortex (Lau and Passingham, 2006; Bang and Fleming, 2018; Gherman and Philiastides, 2018; Shekhar and Rahnev, 2018; Bang et al., 2020; Yeon et al., 2020). Note that confidence here reflected the degree to which one was certain a stimulus was present and is therefore strongly linked to stimulus visibility, which previous studies have shown to be encoded in frontal regions (Mazor et al., 2022). Alternatively, this signal could reflect other processes related to detecting a presence signal, including attentional processes or sensory evidence accumulation. However, for the present study, the important point is that these frontoparietal processes were shared between veridical and false percepts, rather than being distinct. Future studies are needed to disentangle competing interpretations of what exactly drives the confidence signal.

In conclusion, the present study revealed that the neural signals underlying false and veridical perception are largely shared, both at the level of content-specific sensory signals and confidence in stimulus presence. Unique to false percepts, increased high alpha/low beta power arising from a parietal-occipital network specifically preceded high confidence false percepts, but not veridical perception. Thereby, the current study sheds light on how false percepts arise from basic sensory mechanisms causing internal sensory signals to be mistaken for reality.
